# Augmented Reality in Enhancing Operating Room Crisis Checklist Adherence: Randomized Comparative Efficacy Study

**DOI:** 10.2196/60792

**Published:** 2025-01-06

**Authors:** Rayan Ebnali Harari, Abdullah Altaweel, Erik Anderson, Charles Pozner, Rafael Grossmann, Andrew Goldsmith, Hamid Shokoohi

**Affiliations:** 1 STRATUS Center for Medical Simulation, Mass General Brigham, Harvard Medical School, 10 Vining, Boston, MA, 02135, United States, 1 6173721022; 2 Kuwait Ministry of Health, Kuwait City, Kuwait; 3 Department of Emergency Medicine, Mass General Brigham, Boston, MA, United States; 4 PC Institute for Medical Education, Boston, MA, United States; 5 Department of Surgery, Portsmouth Regional Hospital, Boston, MA, United States

**Keywords:** augmented reality, operating room, crisis checklist, checklist, guideline adherence, quality improvement, patient safety, cardiac arrest, hypotension, hyperthermia, critical care, emergency department

## Abstract

**Background:**

Effective crisis management in operating rooms (ORs) is crucial for patient safety. Despite their benefits, adherence to OR crisis checklists is often limited, highlighting the need for innovative solutions.

**Objective:**

The objective of this study was to evaluate the efficacy of augmented reality (AR)-enhanced checklists in improving protocol adherence, compared to traditional paper checklists and no checklist scenarios during simulated OR crises.

**Methods:**

This study was a randomized comparative efficacy study comparing the utility of AR checklists, paper checklists, and no checklist scenarios using 4 validated and simulated OR crises scenarios: asystolic cardiac arrest, air embolism, unexplained hypotension/hypoxia, and malignant hyperthermia. The study took place in a simulated OR setting and had applicability to the standard procedures in ORs, critical care units, and urgent care scenarios in the emergency department. To form the 24 OR teams, 50 professionals including 24 anesthesiologists, 24 nurses, 1 surgeon, and 1 scrub nurse from two academic hospitals were included. The primary outcome measured was the failure to adhere (FTA) rate for critical actions during simulated OR crises. Adherence was determined using retrospective video analysis involving 595 key processes evaluated across 24 surgical teams. Interrater reliability was assessed using a Cohen κ. Secondary outcomes included checklist usability and cognitive load, as measured by the low-frequency to high-frequency (LF/HF) ratio of the heart rate variability.

**Results:**

The AR checklist group showed a significantly lower FTA rate (mean 15.1%, SD 5.77%) compared to the paper checklist (mean 8.32%, SD 5.65%; *t*_23_=−2.08; *P*=.048) and the no checklist groups (mean 29.81%, SD 5.59%; *t*_23_=−6.47; *P*<.001). The AR checklist also resulted in a higher LF/HF ratio for anesthesiologists (*F*_2,46_=4.88; *P*=.02), showing a potential increase in the level of cognitive load. Survey data indicated positive receptions for both AR and paper checklists.

**Conclusions:**

These results suggest that AR checklists could offer a viable method for enhancing adherence to critical care protocols. Although, further research is needed to fully assess their impact on clinical outcomes and to address any associated increase in cognitive load.

## Introduction

Unexpected crises in the operating room (OR), such as cardiac arrests or severe hemorrhages, create a critical situation in which surgical teams should deliver rapid and coordinated care with a time-sensitive order of actions listed in the OR crisis checklists [1-3]. Although these high-stakes, low-frequency crises may occur infrequently for any single practitioner, their cumulative incidence across hospitals underscores a significant challenge to patient safety and surgical outcomes [4-7]. The OR teams’ ability to effectively manage these life-threatening complications depends on their preparedness in managing crises [8,9], training [10], and adherence to the validated crisis checklists [11]. Presurgical checklists are used before surgery to ensure correct patient identification and procedure planning. In contrast, crisis management checklists guide surgical teams during emergencies, helping them respond quickly to life-threatening situations. While both checklists improve safety, this study focuses specifically on crisis management checklists, which aim to support decision-making during critical events in the OR.

The lack of adherence to the checklists negatively impacts surgical mortality rates and overall hospital performance [12]. Evidence suggests that adherence to established best practices during these critical moments is varied and often associated with a decay in the retention of essential skills and knowledge over time [13-16]. In many instances, the use of surgical safety checklists was associated with a reduction in morbidity and mortality, and they were integrated as a new standard of care [17,18]. The dynamic and high-pressure nature of surgical emergencies requires not only adherence to protocols but also the ability to quickly access and use complex information under cognitively demanding conditions [19-21]. However, even though adherence to these checklists is crucial, the traditional paper ones are often difficult to use effectively in such intense scenarios [22-24]. The low adoption of checklists underscores the need for innovative approaches to using checklists that fit with surgical workflows, enhancing protocol adherence without disrupting the clinical focus.

Augmented reality (AR) technology, by relaying important procedural information directly into the clinicians’ vision [25-28], can enhance protocol adherence in medical settings [29-33]. Initial applications of AR in medication management and emergency trauma care have shown promise in reducing errors and guiding clinicians through complex procedures with enhanced clarity and efficiency [34-38]. This evidence positions AR as a potential technology for improving adherence to medical protocols [39-41]. However, the effectiveness of and adherence to AR-enhanced surgical checklists during OR crises has not been thoroughly studied.

This study aims to evaluate the efficacy of AR-enhanced checklists in improving protocol adherence by surgical teams during simulated OR crises. By comparing outcomes with the traditional paper checklists and scenarios without a checklist, the research seeks to provide evidence on AR’s utility to reduce the failure to adhere (FTA) rate for crucial procedural steps when managing surgical crises, ultimately improving patient outcomes in the OR. We hypothesize that the AR-enhanced checklists will significantly reduce the FTA rate for crucial procedural steps compared to traditional paper checklists and no checklist scenarios.

## Methods

### Study Design

**Figure 1.**
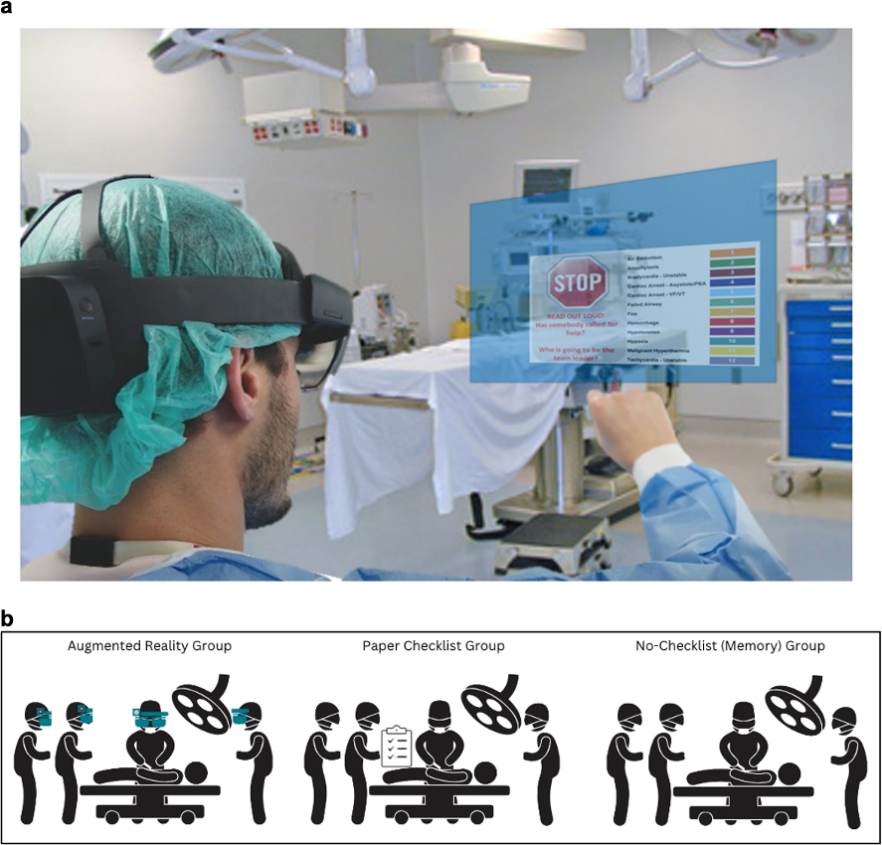
Study overview diagram. (a) Checklists presented in an augmented reality interface using Microsoft HoloLens 2. (b) Study design scenarios including an augmented reality checklist, paper checklist, and no checklist.

This prospective within-subject study aimed to compare the impact of AR checklists, traditional paper checklists, and no checklist conditions on managing OR crises (Figure 1). A detailed outline of team participation and the methodological framework is included in Multimedia Appendix 1. The development and rationale behind the crisis checklists, guided by surgical safety standards, have been detailed in a previous publication [14]. Teams, including anesthesia staff, OR nurses, and a mock surgeon, faced simulated intraoperative crises with randomized scenario assignments and checklist types. Before the main investigation, a pilot study tested the scenario fidelity and the AR checklist’s practicality. Paper checklists were provided in booklet form and placed near the anesthesia machine and the circulating nurse’s station, mirroring their accessibility in actual ORs. A summary and the checklists are available in sections 1‐3 of Multimedia Appendix 1.

### Setups: The OR Checklists

We used OR crisis checklists for 4 critical scenarios: (1) asystolic cardiac arrest, (2) air embolism, (3) unexplained hypotension/hypoxia, and (4) malignant hyperthermia. These scenarios were derived from a comprehensive checklist development and testing process explained by Ziewacz et al [42] and were chosen for their clinical importance and feasibility for implementation in AR. Additionally, we followed the standardized approach used by Arriaga et al [14], which evaluated the efficacy of these checklists in improving adherence to lifesaving protocols through high-fidelity medical simulations. More details on the checklists and key processes evaluated to measure adherence to protocols can be found in section 3 of Multimedia Appendix 1.

### Participants

Participants were recruited from 2 academic hospitals between October 2021, and September 2023. Each team comprised the anesthesia staff (including attending physicians and residents), OR nurses, one mock surgeon, and one scrub nurse, totaling 24 attending physicians and residents, 24 OR nurses, and one mock surgeon across 24 teams. Team formations were randomized. Each team dedicated an average of 3.5 hours within a single day to participate in a high-fidelity simulated OR environment. In the simulated OR, they encountered a series of crisis scenarios designed to test their adherence to critical and evidence-based practices. Recruitment of staff members was facilitated through sign-up sheets and random selection from those scheduled to work on designated study dates. Hospital departments arranged for staff to attend the simulation sessions instead of their regular workday. Hospital or department rules required that all anesthesia staff taking part had to have up-to-date certification in advanced cardiac life support. Each participant only took part in one study session.

### Ethical Considerations

Ethical approval for this study was obtained from the Ministry of Health, Kuwait (IRBl: SKU-219328). Informed consent was obtained from all participants prior to their involvement in the study. Participants were informed about the study's objectives, procedures, and their rights, including the ability to withdraw at any point without any repercussions. All data collected during the study were deidentified and stored securely to ensure participant confidentiality. Data were anonymized during analysis to protect privacy, and access was restricted to authorized personnel only. No monetary or nonmonetary compensation was provided to participants for their involvement in this study. Identifiable features of participants were not captured in any images or supplementary materials.

### Primary Outcome: FTA rate

The primary outcome was the FTA rate for 47 key lifesaving processes outlined in Multimedia Appendix 1. Adherence was evaluated and scored as either yes or no by 2 physician reviewers from our team (AA and RG) who observed and scored recorded simulation sessions. These sessions were recorded as synchronized videos on 2 screens for a comprehensive review. To ensure the accuracy of adherence scoring, interrater reliability was assessed. Any disagreements or uncertainties in scoring were reviewed by third reviewers (CP, HS) and were resolved. The primary variables included the checklist group and the medical crisis scenario. The primary aspect of the study was the measured FTA rates.

### Secondary Outcomes

#### Cognitive Load

We used a Polar chest strap to collect interbeat interval data from participants during scenarios with an accuracy of 1 millisecond. Previous studies have shown that a low-frequency to high-frequency (LF/HF) ratio extracted from heart rate variability is a validated proxy for cognitive load [43-45], particularly when collected using chest wraps [46]. We used NeuroKit2, a toolbox for neurophysiological signal processing [47], to extract the LF/HF ratio from data aggregated into a 1-minute time window.

#### Participant Satisfaction and Usability

To evaluate the ease of use and the perceived effectiveness of the AR and paper checklists, we administered a structured survey adopted from Arriaga et al [14]. The survey assessed participants’ preparedness, ease of use, readability, willingness to use the checklist in real scenarios, and perceived impact on the clinical flow during emergencies. Responses were captured on a Likert scale ranging from 1 (strongly disagree) to 5 (strongly agree), providing insights into participants’ attitudes and perceptions across various aspects of checklist usage.

### Statistical Analysis

Participant characteristics were presented by descriptive statistical analysis, which reported the number and percentage of participants across different roles and years of experience. To assess the consistency in observational scoring, the agreement between two reviewers on the adherence scores was quantified using a Cohen κ. The Shapiro-Wilk test was used to evaluate the normality of the data distribution. ANOVA was used to compare the efficacy of interventions across 3 groups and post hoc analyses were conducted to examine the checklist’s efficacy across various scenarios. Participant satisfaction and usability were analyzed using descriptive statistics and reporting means and SD. The statistical analyses were performed using SAS with all *P* values being 2-sided and a threshold for statistical significance set at *P*<.05.

## Results

### Participants

A total of 50 participants, forming 24 teams, took part in this study, which included anesthesiologists (n=14), anesthesia residents (n=10), OR nurses (n=24), a surgical resident (n=1), and a scrub nurse (n=1). All anesthesia residents were in the early stages of their careers with 0‐2 years of experience, and OR nurses included a more diverse range of experience, spanning from 0‐8 years. Each team contained 1 mock surgeon and 1 surgical assistant (scrub nurse), who attended as stand-in participants to the operative field without participating in decision-making or survey completion; these stand-in staff members were not counted as participants. Participants’ years of experience are summarized in Table 1.

**Table 1.** Participant’s role and their years of experience.

Role		Years of experience in specialty, n (%)			
		0‐2	2‐8	>8	Unknown
Anesthesiologist					
Attending physician (n=14)		0 (0)	7 (50)	7 (50)	0 (0)
Anesthesia resident (n=10)		10 (100)	0 (0)	0 (0)	0 (0)
Operating room nurse (n=24)		6 (25)	12 (50)	3 (12.5)	3 (12.5)
Surgical resident (n=1)		(1) 100	0 (0)	0 (0)	0 (0)
Scrub nurse (n=1)		0 (0)	1 (100)	0 (0)	0 (0)

### Adherence Rating

The assessment of adherence to key processes during the simulated scenarios demonstrated high interrater reliability among independent reviewer pairs, with Cohen κ values of ≥0.83 across all pairs. In instances where initial disagreement or uncertainty arose among the physician reviewers, consensus was reached through expert review with video replay. Out of a total of 595 key processes, evaluated across 24 teams for 25 key processes (excluding 8 key processes from one team that did not initiate the unexplained hypotension/hypoxia followed by an unstable bradycardia scenario), only 23 instances necessitated this expert review. The process of video replay facilitated immediate full agreement among all reviewers, highlighting the effectiveness of this approach in resolving ambiguities and ensuring accurate adherence assessment.

### Comparing Groups Across All 4 Crisis Scenarios

**Figure 2.**
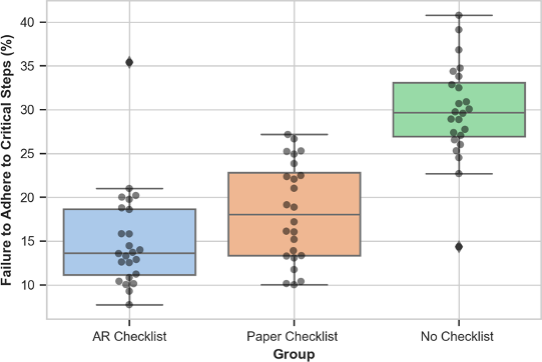
Failure to adhere to critical steps by condition type.

ANOVA analysis showed significant differences in the FTA rate for critical steps among the 3 checklist groups (*F*_2,46_=48.3; *P*<.001). Subsequent post hoc analysis showed the AR checklist group’s mean FTA rate of 15.1% (SD 5.77%, 95% CI 13.50-16.70) was significantly lower than the paper checklist group’s FTA rate of 18.32% (SD 5.65, 95% CI 16.75-19.89) and the no checklist group’s FTA rate of 29.81% (SD 5.59, 95% CI 28.26-31.36). The AR group’s FTA rate was significantly less than the no checklist group (*t*_23_=−10.9; *P*<.001) and the paper checklist group (*t*_23_=−2.08; *P*=.048). Moreover, the paper checklist group also had a significantly lower FTA rate compared to the no checklist group (*t*_23_=−6.37; *P*<.001; Figure 2).

### Comparing Groups for Individual Crisis Scenarios

Adherence to critical steps across various scenarios demonstrated significant differences among groups, with an ANOVA test showing distinct results for asystolic cardiac arrest (*F*_2,46_=25.07; *P*<.001), air embolism (*F*_2,46_=14.90; *P*<.001), malignant hyperthermia (*F*_2,_

**Figure 3.**
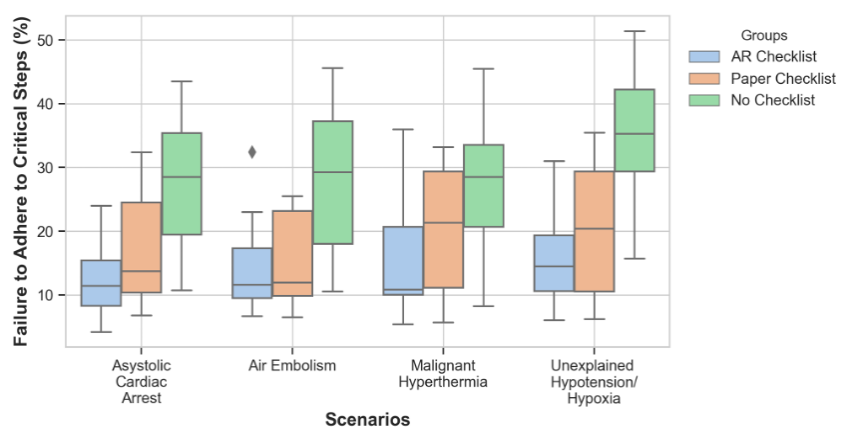
Failure to adhere to critical steps by scenario and group type. AR: augmented reality.

_46_=12.33; *P*<.001), and unexplained hypotension/hypoxia (*F*_2,46_=38.39; *P*<.001). Post hoc analyses indicated that, across these scenarios, the AR checklist group consistently exhibited significantly lower FTA rates compared to the no checklist group, with notable differences in asystolic cardiac arrest (*t*_23_=−6.47; *P*<.001), air embolism (*t*_23_=−4.45; *P*<.001), malignant hyperthermia (*t*_23_=−4.79; *P*<.001), and unexplained hypotension/hypoxia (*t*_23_=−10.57; *P*<.001). Comparisons between the AR and paper checklist groups were only significant for some scenarios, with slightly lower FTA rates for critical steps using the AR checklist in asystolic cardiac arrest (*t*_23_=−2.65; *P*=.014) and unexplained hypotension/hypoxia (*t*_23_=−2.10; *P*=.046). The paper checklist group also demonstrated significantly improved adherence over the no checklist condition in scenarios such as an air embolism (*t*_23_=3.72; *P*<.001) and unexplained hypotension/hypoxia (*t*_23_=5.40; *P*<.001; Figure 3).

While the AR checklist group demonstrated statistically significant differences in FTA rates compared to the paper checklist group, it is important to note that this significance was observed by a narrow margin. Given the sample size, there remains the possibility that this effect could be influenced by chance, and further studies with larger sample sizes are necessary to confirm these findings.

### Cognitive Workload

**Figure 4.**
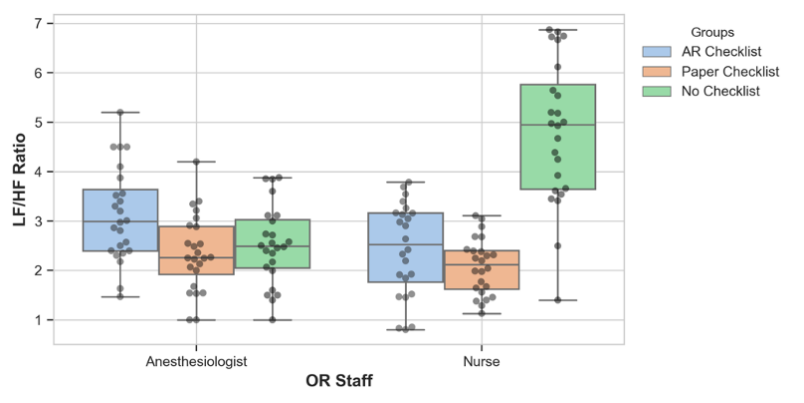
Low-frequency to high-frequency ratio across operating room staff roles by checklist group. AR: augmented reality; LF/HF: low frequency to high frequency; OR: operating room.

For anesthesiologists, ANOVA results showed a significant effect of the checklist type on the LF/HF ratio (*F*_2,46_=4.88; *P*=.02). In pairwise comparisons, the AR checklist group had a significantly higher LF/HF ratio compared to both the paper checklist and no checklist groups, suggesting a potential increase in cognitive load when using the AR checklist (*P*<.05; Figure 4). There was no significant difference in LF/HF ratio when comparing the paper checklist with no checklist groups, after adjusting for multiple comparisons. For nurses, the differences were significantly different (*F*_2,46_=43.25; *P*<.001). The no checklist group had a significantly higher LF/HF ratio than the other two groups (*P*<.05). The AR checklist and paper checklist groups did not differ significantly.

### Survey

Survey responses showed that both AR and paper checklist groups viewed their respective checklists positively (Table 2). Participants in the AR checklist group rated the checklist’s ability to help them feel prepared during the emergency scenario at a mean Likert score of 4.5 (SD 0.75), and the paper checklist group rated this at 4.3 (SD 0.82), indicating no significant difference between the groups. Participants expressed a strong willingness to use the checklists in real-life situations, with the AR group scoring a 4.6 (SD 0.70) and the paper group scoring a 4.4 (SD 0.75). When considering the disruption to the clinical flow of the operative emergency, the AR checklist group reported less disruption with a mean score of 4.5 (SD 0.90) compared to the paper checklist group’s score of 4.2 (SD 1.00).

**Table 2.** Questionnaire response data from participants on checklist usability.

Statement	AR^a^ checklist group (n=48), mean (SD)	Paper checklist group (n=48), mean (SD)	*P* value
The checklist helped me feel better prepared during the emergency scenario.	4.5 (0.75)	4.3 (0.82)	.13
The checklist was easy to use.	4.4 (0.80)	4.2 (0.85)	.09
I would use this checklist if I were presented with this operative emergency in real life.	4.6 (0.70)	4.4 (0.75)	.03
The checklist did not disrupt the clinical flow of the operative emergency.	4.5 (0.90)	4.2 (1.00)	.04
If I were having an operation and experienced this intraoperative emergency, I would want the checklist to be used.	4.7 (0.55)	4.6 (0.60)	.18

a AR: augmented reality.

## Discussion

### Principal Findings

Our findings show that AR checklist groups had a superior adherence to critical steps in crises when compared to the paper checklist groups and groups who did not use any checklist. These findings highlight AR’s potential to improve OR staff’s adherence to predefined protocols and ultimately improve patient outcomes. This improvement suggests that sending critical and time-sensitive information to clinicians’ and OR staff’s field of view may help with faster and more precise decision-making in critical situations and emergencies. Considering a day-by-day improvement in technology, this will have the potential to set the ground for an extended and more effective AR checklist intervention in many other critical scenarios. This potential benefit is in line with a comparison of the AR checklist versus the traditional checklist in other health care applications [29,30]. The benefit of AR checklists, particularly in comparison with non-AR alternatives, underscores the technology’s capacity to augment traditional safety measures.

It is also important to note that while the AR checklist group had a clear superiority over the no checklist group, the margin of improvement was modest when it was compared to the paper checklist group. In this comparison, the differences were not always statistically significant across different scenarios. These findings suggest that AR technology may not offer the same improvement in all clinical scenarios over the paper checklists. Considering the low sample size and extensive subgroup analysis, it is reasonable to suggest that AR’s real-world application and its superiority over conventional methods warrant further examination. We also observed variation in team performance, as highlighted in Figure 1 of Multimedia Appendix 1. Some of this variation may be attributed to an order effect, where teams became more familiar with the simulation environment over time. This potential bias should be considered when interpreting the results, and future studies could include randomization or counterbalancing to mitigate this effect.

The feedback from participants indicated a high level of acceptance and perceived utility of AR checklists in crisis scenarios, pointing to the potential for AR to integrate effectively into surgical workflows. However, the nuanced performance improvements highlight the need for a tailored approach to technological integration in health care, where the specific context and user needs dictate the effectiveness of such alternatives [48-50]. The study’s results align with broader trends in medical and high-risk industries, where checklists have long been recognized for their role in promoting adherence to best practices and enhancing outcomes [51-53]. Just as checklists have transformed safety protocols in aviation and nuclear power, AR checklists hold promise for surgical settings. Nonetheless, the adaptation of these tools in medicine, particularly in the high-stakes environment of the OR, requires careful consideration of design, implementation, and training to ensure they meet the unique demands of health care providers and patients.

A key consideration emerging from our research is the differential impact of AR on the cognitive load among OR staff. Anesthesiologists using the AR checklist have shown a higher LF/HF ratio, which may be associated with a higher level of cognitive load when compared to the paper and no checklist groups. While we initially interpreted the higher LF/HF ratio in the AR checklist group as a sign of increased cognitive burden, it is also possible that this reflects heightened cognitive engagement. The AR checklist may stimulate more focused attention on the OR environment and monitoring, compared to the paper checklist, which could be perceived as more distracting. This alternative interpretation suggests that the AR condition may enhance attentional focus in a high-stakes environment, and further research is needed to clarify the relationship between LF/HF ratio and cognitive engagement.

It is an important finding that AR technology may improve adherence but simultaneously may add a cognitive burden [54,55] that adversely affects clinicians’ behavior under cognitively demanding conditions. This variability in cognitive impact across different OR roles underscores the importance of designing AR applications that are tailored to the diverse needs and cognitive capacities of surgical teams. Future studies should also include qualitative methods to capture participants’ experiences with AR and paper checklists. Combining this with quantitative data will provide a more complete understanding [56].

### Limitations

This study has several limitations that should be considered. First, the study was conducted in a simulation setting that may not necessarily reflect the complexity of the OR environment. Second, our sample size was relatively small with a limited statistical power that prevented us from confidently performing subcategory analysis and extracting minor differences between groups. Larger studies with more diverse groups of clinicians and more scenario variability are needed to allow for subgroup analyses and to look for potential impacts on certain groups of clinicians or crisis scenarios. Third, the integration of AR technology into clinical practice raises questions about cost, accessibility, and the need for specialized training [57]. The development of best practices for the implementation and customization of AR checklists will be crucial to their successful adoption in surgical care. Last, we recognize that *P* values alone should not be taken as conclusive evidence of AR’s superiority. The narrow statistical margin highlights the need for further validation through larger studies to confirm its efficacy.

### Conclusion

Our study showed that the use of AR-enhanced checklists significantly improved adherence to critical procedural steps during simulated OR crises compared to both traditional paper checklists and scenarios without a checklist. These findings are promising as they may contribute to the patient’s safety and outcomes. However, while the benefits of AR are promising, our findings also indicate a potential increase in cognitive load among clinicians, particularly anesthesiologists. Future studies should aim to optimize AR interfaces to minimize cognitive demands and validate these results in real-world settings. Addressing the balance between improved protocol adherence and cognitive load will be crucial for integrating AR effectively in high-stakes environments like the OR.

## Supplementary material

10.2196/60792 Multimedia Appendix 1 Supplementary materials on the development and application of augmented reality checklists for crisis management in clinical settings.

## Abbreviations

AR: augmented reality
FTA: failure to adhere
LF/HF: low-frequency to high-frequency
OR: operating room
