# Supplementary material for: Augmented Reality in Enhancing Operating Room Crisis Checklist Adherence: Randomized Comparative Efficacy Study
Source: JMIR XR Spat Comput. 2025 Jan 6;2:e60792. doi: 10.2196/60792 (PMC13202501; doi:10.2196/60792)
Supplement: Multimedia Appendix 1 [file xr-v2-e60792-s001.docx]

**Supplementary Materials**

**Introduction**

These supplementary materials elaborate on the development and application of augmented reality (AR) checklists for crisis management in clinical settings, including the methodology behind checklist creation, the content of each checklist, the technical development process of the AR system, and the sequencing of medical crisis scenarios, covered from part 1 to 7.

1. **Checklist methodology**

The checklists materials were adopted from a previous study in which they tested checklists for 12 medical crises. As noted in the manuscript, a detailed description of the development and pilot testing of the crisis checklists can be found here (Arriaga AF, Bader AM, Wong JM, et al. Simulation-based trial of surgical-crisis checklists. N Engl J Med 2013;368:246-53. DOI: 10.1056/NEJMsa1204720).

Following a previously established methodology, we adopted 4 critical checklists. Then based on the paper-based checklist, we created AR version which exactly followed the same protocol and contained the same content as paper version included and provided a digital overlay of content to AR HMD. We then conducted pilot testing to ensure the feasibility of the AR checklists.

This resulted in the development of four AR checklists with triggers for key life-saving evidence-based processes of care for each crisis. After the initial checklist development, minor modifications were made based on updates to national consensus guidelines and lessons learned from early pilot testing.

About one-third of the scenarios provided teams access to a paper checklist, another third to an AR checklist (showing the same information as the paper one but using an AR interface), and the last third did not provide any checklist, so teams had to rely on memory, like usual practices. The allocation and sequence of scenarios and checklist modalities were randomized to reduce biases in assessment.

1. **Content of checklists:**

Four of these checklists were selected to assess the efficacy of AR checklists, versus paper checklists, versus no checklist in medical crises: Air embolism, unstable bradycardia, asystolic cardiac arrest, hypotension, hypoxia, malignant hyperthermia (Arriaga et al. 2013).

#### **AR development**

The development of the AR version of the checklists involved an iterative approach, utilizing Unity software and the Mixed Reality Toolkit (MRTK) for design and implementation, and then we tested it on Microsoft HoloLens 2 headsets. This process began with the identification of essential life-saving procedures from existing validated crisis management checklists. The goal was to ensure the AR checklist covered all the critical aspects needed for effective crisis response in the OR, based on evidence-based guidelines.

During the design phase, we focused on creating an intuitive user interface that could be easily integrated into the OR practice without disrupting workflow. We chose Unity, a cross-platform game engine, as it is versatile and adaptable in developing all sorts of AR experiences. MRTK provided a set of tools and features designed for building AR applications, facilitating the development of an interactive checklist. The application was then deployed on Microsoft HoloLens 2 headsets, selected for their AR capabilities and the ability to project digital information onto the real world in a hands-free manner, which is crucial for maintaining sterile conditions and accessing information quickly. Microsoft HoloLens 2 headset has already cleared FDA approval for preoperative surgical planning[^18^](https://paperpile.com/c/BGGLqH/aDvc).

Before its integration into the main study, we studied the AR checklist with four individuals who were not part of the main experiment. This preliminary evaluation aimed to assess the checklist' integration into clinical practice. Feedback from these participants highlighted areas for improvement, such as adjusting the visual presentation of the checklist for easier readability under various lighting conditions. The insights gained from this pilot testing were used in refining the AR checklist.

1. **Scenarios sequencing**

The following describes the design of our study regarding sequences of situations that our groups of participants managed their simulated medical crises, and the 25 key processes tracked in our study.

**Table 1.** Sequence of medical crises by type and checklist used

| Key | Crisis Number | | 1 | | 2 | | 3 | | 4 | |
| --- | --- | --- | --- | --- | --- | --- | --- | --- | --- | --- |
|  |  |  | Asystolic Cardiac Arrest | | Air Embolism | | Unexplained Hypotension/Hypoxia Followed by Unstable Bradycardia | | Malignant Hyperthermia | |
|  | Checklist used by color | | No Checklist | | Paper Checklist | | AR Checklist | | NA | |
| Sequence of scenarios by type of event and checklist | | | | | | | | | | |
| Sequence 1 | | Sequence 2 | | Sequence 3 | | Sequence 4 | | Sequence 5 | | Sequence 6 |
| 1 | | 2 | | 4 | | 3 | | 2 | | 4 |
| 4 | | 3 | | 1 | | 2 | | 1 | | 2 |
| 3 | | 1 | | 2 | | 4 | | 3 | | 1 |
| 2 | | 4 | | 3 | | 1 | | 4 | | 3 |

1. **Key processes tested (adopted from Arriaga et al. 2013)**

***5.1 Key Processes for Asystolic Cardiac Arrest:***

1. Chest compressions are initiated within one minute of onset of asystole or pulseless electrical activity (PEA).

2. After onset of asystole/PEA, chest compressions (once initiated) are given without prolonged interruption(s) (No pause greater than 30 seconds).

3. Patient does not receive shock while pulse/rhythm indicates asystole/PEA.

4. Initial dose of epinephrine (or vasopressin) given within 3 minutes of onset of asystole/PEA.

5. Repeat dose of epinephrine (or vasopressin) given within 3-5 minutes after the first dose.

6. At least one team member in the room explicitly calls for outside help (e.g. phone call) within 1 minute of onset of Asystole/PEA.

7. At least one member reads aloud the H’s and T’s (or explicitly discusses the causes in any order) within 10 minutes of the start of asystole/PEA.

***5.2 Key Processes for Air Embolism:***

8. Fi02 increased to 100% within 3 minutes of air embolism (indicative signs: significantly decreased end-tidal CO2 and oxygen

desaturation).

9. Attempts made to stop the source of air entry within 5 minutes of air embolism (Scenario specific.

10. At least one team member explicitly call for help (e.g. phone call) within 3 minutes of the onset of air embolism.

***5.3 Key Processes for Unexplained Hypotension/Hypoxia followed by Unstable Bradycardia:***

11. IV fluids opened wide or fluid bolus given within 3 minutes of unstable hypotension.

12. FiO2 increased to 100% within 3 minutes of unstable hypotension/hypoxia.

13. Hand Ventilation initiated within 3 minutes of hypoxia.

14. Breath sounds auscultated within 3 minutes of hypoxia.

15. Suction provided through the endotracheal tube.

16. At least one team member in the room explicitly calls for outside help (e.g. phone call) within 3 minutes of onset of unstable hypotension/hypoxia.

17. Atropine given within 5 minutes of unstable bradycardia.

18. Transcutaneous pacing established within 3 minutes of unstable bradycardia onset.

**5.4 Key Processes for Malignant Hyperthermia:**

19. Dantrolene given within 7 minutes of MH.

20. All volatile anesthetics stopped within 3 minutes of onset of MH (increased ETCO2, tachycardia, febrile).

21. FiO2 increased to 100% within 3 minutes of onset of MH

22. Patient hyperventilated (i.e. 2-4 times the minute ventilation the patient was initially receiving) within 3 minutes of onset of MH.

23. Attempts made to cool the patient within 5 minutes of MH (cooling blanket, gastric lavage, and/or external ice packs).

24. Drugs given to treat hyperkalemia within 5 minutes of rhythm change.

25. At least one team member in the room explicitly calls for outside help (e.g. phone call) within 3 minutes of onset of malignant hyperthermia.

1. **Team participation**

Each of the 24 teams participated in one unique sequence. A crossover design was used (i.e. each team randomly performed their scenarios with the paper, AR checklists or no checklist available) to reduce potential spillover effects of teams using the checklists in an alternating fashion. Each team contained 1 mock surgeon and 1 surgical assistant (scrub nurse), who attended the operative field without participating in decision-making or completing surveys; these stand-in staff members were not counted as participants).

The selection of checklist was based on feasibility of implementation in a simulation-based OR environment. For example, failed airways and fire were not simulated due to resource constraints, as well as a need to limit the length of the study day for a given participating team. The randomization of the scenario order and checklist availability was performed using a random number generator in Microsoft Excel 2007. To avoid an overabundance of the same type of crisis in either the intervention or control group, the order of these scenarios were randomized such that each team managed one of each scenario type (i.e. one cardiac arrest, one case of unstable bradycardia or tachycardia, and one non-ACLS case [anaphylaxis or

malignant hyperthermia]) with the checklists available and a different one of each type by memory alone.

1. **Additional results:**

**Differential team performance:** Adherence across teams: The distribution of failure rates varies widely between teams, with some demonstrating as low as approximately 10% and others peaking near 40%. The median failure rate for most teams lies between 20% to 30%, with a few teams showing notably higher or lower medians. Notable outliers are present, indicating instances where teams' failure rates were significantly higher or lower than the typical range. The variability suggests differences in how teams performed with respect to adherence to critical steps, which could be influenced by factors such as experience, familiarity with the checklist system, or the complexity of the scenarios presented to each team. Differential in team performance in Figure 1 underlines the necessity for studying a large number of groups, and the necessity for testing groups on all of the checklist scenarios (AR, paper, and no checklist).


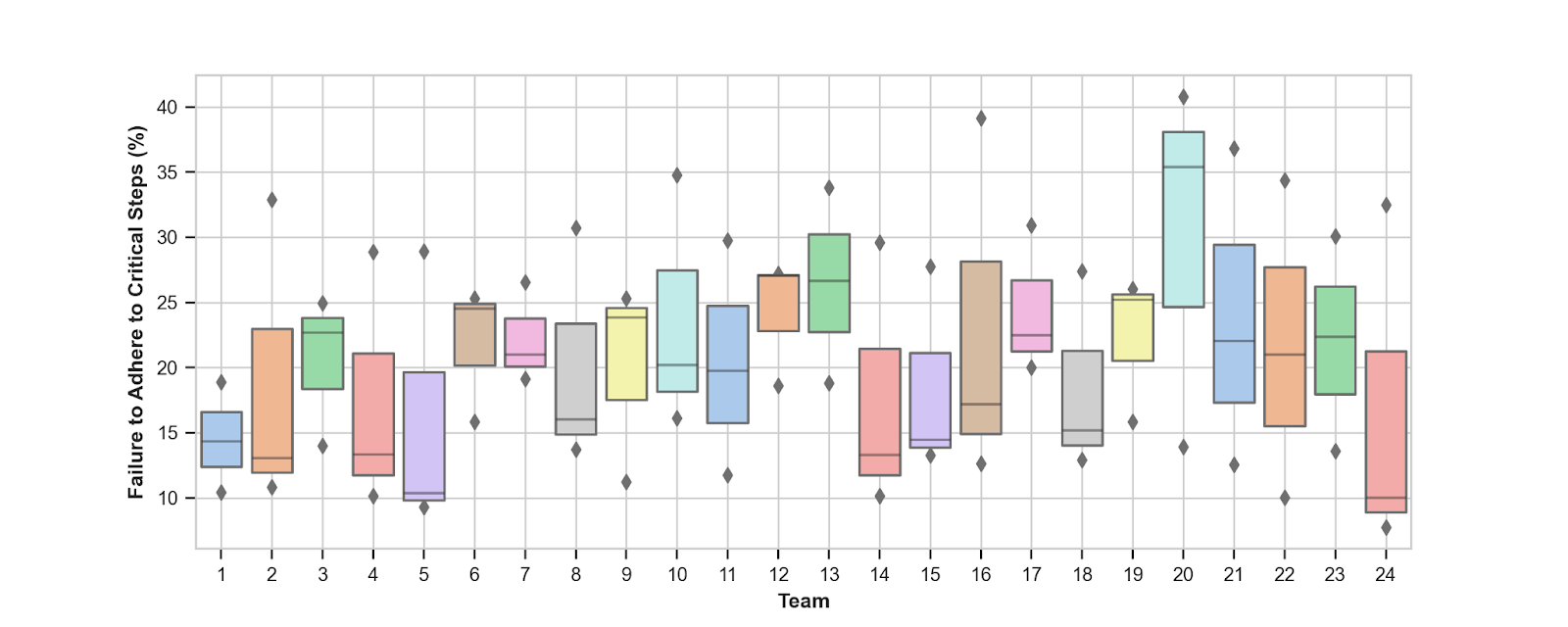


**Figure 1.** Boxplot of failure to adhere to critical Steps (%) across 24 teams in operating room crisis scenarios

1. **References**

Arriaga AF, Bader AM, Wong JM, et al. Simulation-based trial of surgical-crisis checklists. N Engl J Med 2013;368:246-53. DOI: 10.1056/NEJMsa1204720

Devoto, Laurence, Sam Muscroft, and Manish Chand. 2019. “Highly Accurate, Patient-Specific, 3-Dimensional Mixed-Reality Model Creation for Surgical Training and Decision-Making.” *JAMA Surgery* 154 (10): 968–69.
